# Chest wall schwannoma presenting as a solitary malignant lesion: a case report

**DOI:** 10.1186/s40064-016-3270-6

**Published:** 2016-09-13

**Authors:** M. Galukande, A. Khingi

**Affiliations:** 1 International Hospital Kampala, Namuwongo, P.O. Box 8177, Kampala, Uganda; 2 Surgery Department, College of Health Sciences, Makerere University, Kampala, Uganda; 3 International Hospital Kampala, Kampala, Uganda

**Keywords:** Schwannoma, Chest wall, Lesion

## Abstract

**Background:**

Chest wall schwannomas are rare tumors arising from the intercostals nerves. Schwannomas are lobulated, encapsulated spherical masses, different from neurofibromas in that matter. Men and women are equally affected in their third and fourth decades.

**Case presentation:**

A 42 year old female presented with a 6 month history of progressively worsening pain over the right shoulder and chest wall, aggravated by movement and with associated right arm oedema and paraesthesia. She believed mild symptoms began 2 years prior to presentation. The histopathological examination revealed a benign lesion; a schwannoma with degenerative changes. The section revealed a benign nerve sheath tumor characterized by a proliferation of band spindle cells arranged in fascicles and variable cellularity and associated with dilated and think walled blood vessels.

**Conclusion:**

Chest wall schwannomas are rare; they mimic chest wall malignant lesions.

## Background

Chest wall Schwannomas are rare tumors arising from the intercostals nerves (Matsumoto et al. 2009; Ozaki et al. 2004). Schwannomas are lobulated, encapsulated spherical masses, different from neurofibromas in that matter. Men and women are equally affected in their third and fourth decades (Yamaguchi et al. 2004). Usually, they are asymptomatic and benign, and very rarely malignant or multiple (Dabir et al. 1990; Shoji et al. 2005; Kaneko et al. 2008; Cohen et al. 1986). Schwannomas usually arise from a spinal nerve root, indeed they may arise from any other intrathoracic nerve (Yamaguchi et al. 2004; Kaneko et al. 2008). Radiologically they are sharply demarcated with rare calcifications. CT contrast enhanced scan of the chest shows in accordance, a sharply demarcated mass with low densities and mild enrichment, rarely with calcifications and no fat.

## Case presentation

A 42 year old female presented with a 6 months history of progressively worsening pain over the right shoulder and chest wall, aggravated by movement and with associated right arm oedema and paraesthesia. She believed mild symptoms began 2 years prior to presentation.

On physical examination she had a tender mass over the 2nd–5th intercostals spaces with over lying visibly engorged veins. The blood pressure was BP 130/70 mmHg and pulse rate (PR) was 62 bpm and regular.

**Fig. 1:**
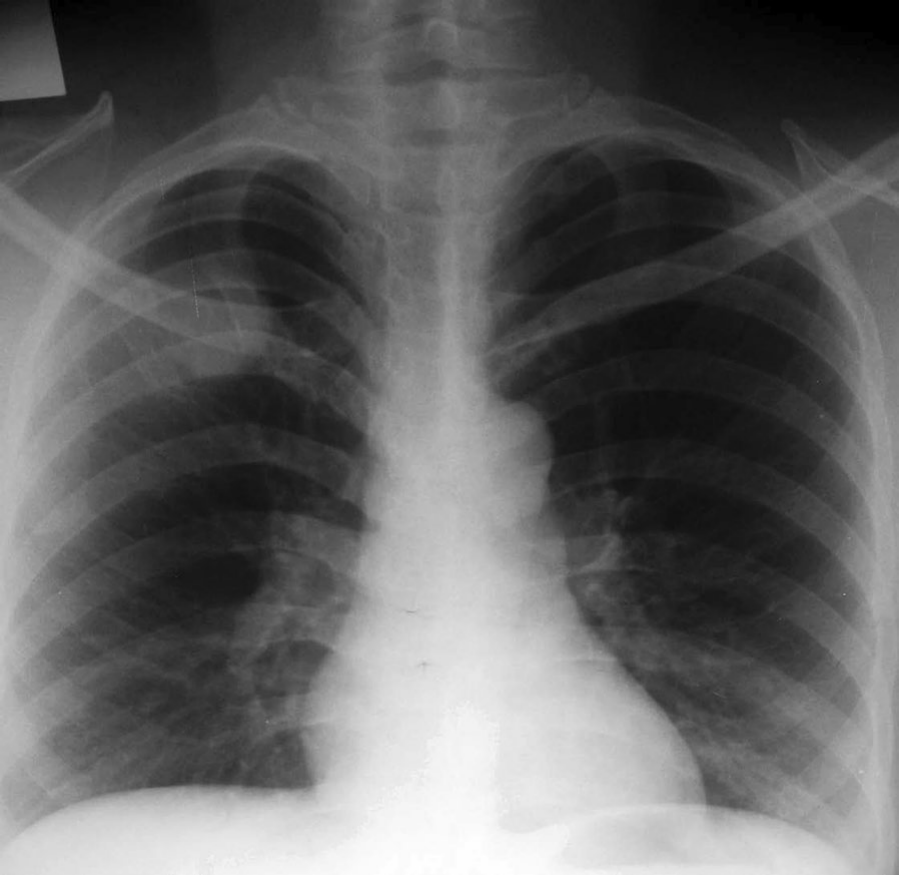
An antero posterior chest X-ray of the patient with a chest wall schwannoma

**Fig. 2:**
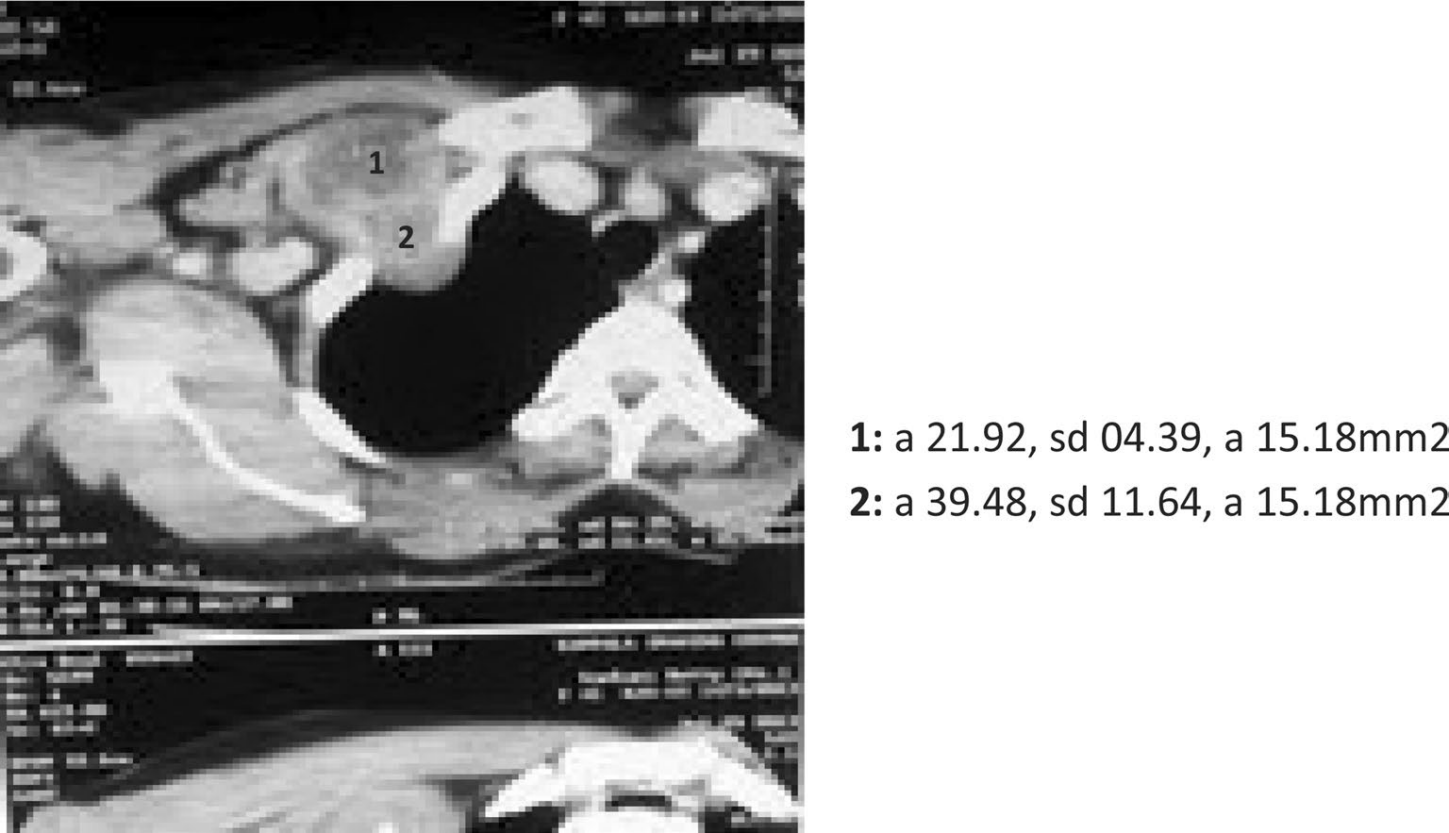
A CT Scan film of a patient with second intercostal nerve schwannoma

A chest xray showed a vague shadow over the 3rd intercostals space mid clavicular line, (Fig. 1), a CT scan showed clearly a mass extending to a butting the pleural cavity pleura, see Fig. 2.

An echocardiogram and ECG were normal; cardiac enzymes panel was normal too.

The patient was consented for an exploration under general anesthesia, in supine position under general anaesthesia (GA), a 6–8 cm incision was made over the mass and a combination of blunt and sharp dissection the mass was wholly excised without opening into the pleura.

The patient was discharged on the 3rd post operation day, pain had significantly reduced and the limb oedema nearly resolved.

The histopathological examination revealed a benign lesion; a schwannoma with degenerative changes. The section revealed a benign nerve sheath tumor characterized by a proliferation of band spindle cells arranged in fascicles and variable cellularity and associated with dilated and think walled blood vessels. There was no evidence of malignancy.

Schwannoma is a benign neurogenic tumour and is usually founded as a solitary lesion. Chest wall schwannoma is rare and usually asymptomatic (Moon et al. 2010). In this patient it was symptomatic and mimicked a metastatic lesion even though no primary lesion was detected prior to the exploratory surgery. The lesion was completely excised under general anaesthesia and endotracheal intubation in supine position. The procedure lasted 45 min with minimal blood loss. The immediate post operative period was unremarkable. A review at 8 weeks after surgery was unremarkable too. Treatment is complete surgical excision, however long term follow up is required in case of recurrence (Chen et al. 2008).

Differential diagnosis includes neurofibroma and leiomyoma. Neurofibromas are not encapsulated and lack the biphasic pattern of schwannomas. Leiomyomas have spindle cells with tapering cytoplasm and elongated blunt ended nuclei (Sawas et al. 2009). This lesion had proliferation of band spindle cells with no evidence of malignancy.

## Conclusion

Chest wall schwannomas are rare; they mimic chest wall malignant lesions.

## Consent

Written and signed informed consent was obtained from the patient for using the pictures and for publication of this information.

## Abbreviations

CT scan: computerized tomograph scan
ECG: electrocardiogram
GA: general anesthesia
PR: pulse rate

## References

[CR1] Chen F, Nakayama E, Okubo K, Date H (2008). Intrathoracic multiple schwannomas of a single intercostals nerve. Ann Thorac Surg.

[CR2] Cohen L, Schwartz A, Rockoff S (1986). Benign schwannomas: pathologic basis for CT inhomogeneities. Am J Roentgenol.

[CR3] Dabir RR, Piccione W, Kittle FC (1990). Intrathoracic tumors of the vagus nerve. Ann Thorac Surg.

[CR4] Kaneko M, Matsumoto I, Oda M, Watanabe G (2008). Multiple schwannoma of the intrathoracic vagal nerve; report of a case. Jap J Thorac Surg.

[CR5] Matsumoto T, Kanzaki M, Wachi N, Onuki T (2009). Surgically treated chest wall schwannoma without entering the pleural space utilizing ultrasonography. Kyobu Geka.

[CR6] Moon H, Park SJ, Kim SR, Park HS, Lee YC (2010). Benign intercostal schwannoma mimicking a solitary metastasis from lung cancer. Thorax.

[CR7] Ozaki S, Miyata Y, Arita M, Takahashi M, Haruta R, Asahara T, Kaneko M, Kurisu K, Kataoka T (2004). Chest wall schwannoma associated with neurofibromatosis 2–a case report. Hiroshima. J Med Sci.

[CR8] Sawas FA, Lababede O, Meziane MA, Arrossi AV (2009). A 54-year old woman with incidentally discovered mass on a chest radiograph. Chest.

[CR9] Shoji F, Maruyama R, Okamoto T, Wataya H, Nishiyama K, Ichinose Y (2005). Malignant schwannoma of the upper mediastinum originating from the vagus nerve. World J Surg Oncol.

[CR10] Yamaguchi N, Yoshino I, Fukuyama S, Osoegawa A, Kameyama T, Tagawa T, Maehara Y (2004). Surgical treatment of neurogenic tumors of the chest. Ann Thorac Cardiovasc Surg.

